# Supplementary material for: NMR Metabolomic Analysis of Skeletal Muscle, Heart, and Liver of Hatchling Loggerhead Sea Turtles (Caretta caretta) Experimentally Exposed to Crude Oil and/or Corexit
Source: Metabolites. 2019 Jan 26;9(2):21. doi: 10.3390/metabo9020021 (PMC6410094; doi:10.3390/metabo9020021)
Supplement: Supplementary file 1 [file metabolites-09-00021-s001.pdf]

Supplementary Materials

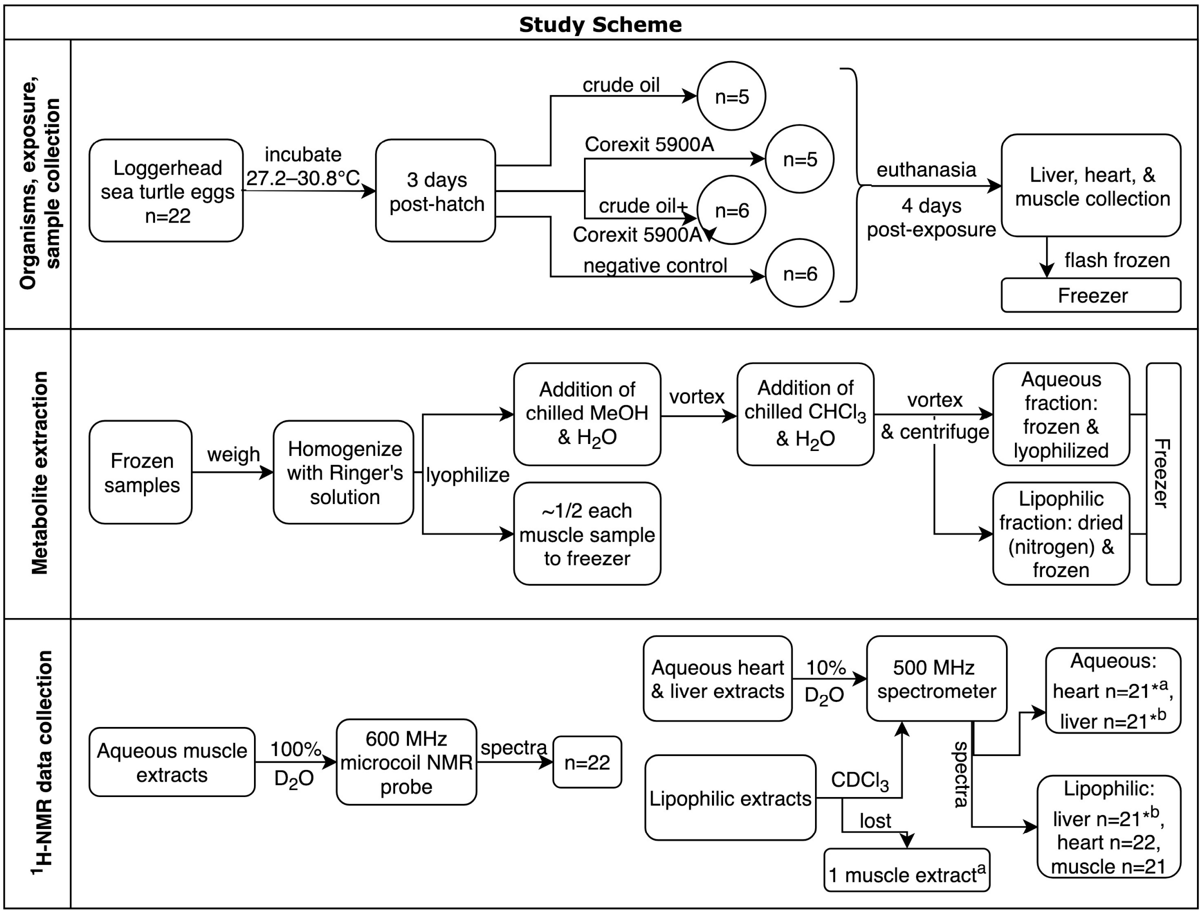

*One spectrum was excluded from data analysis due to poor peak shape and resolution.
[a]Corexit group, [b]negative control group.

Figure S1. Study scheme.

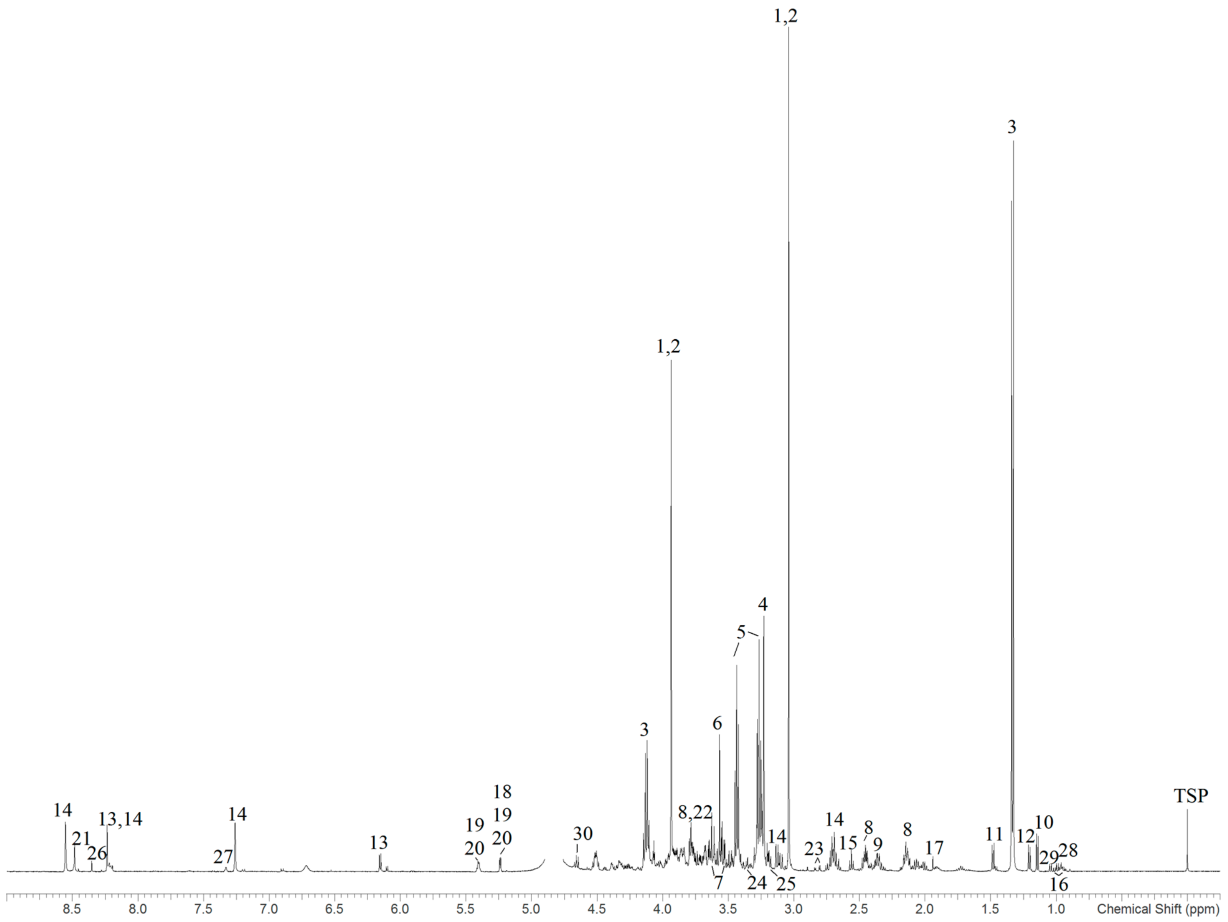

Figure S2. ^{1}H-NMR spectrum of the aqueous extract of combined control samples of hatchling loggerhead sea turtle (*Caretta caretta*) skeletal muscle. The y-axis is indicative of peak intensity, which correlates to concentration. The water peak has been removed. Labeled peaks are as follows: (1) creatine (2) phosphocreatine, (3) lactate, (4) phosphocholine, (5) taurine, (6) glycine, (7) myo-inositol, (8) glutamine, (9) glutamate, (10) propylene glycol, (11) alanine, (12) 3-hydroxybutyrate, (13) inosine monophosphate, (14) carnosine, (15) beta alanine, (16) isoleucine, (17) acetate, (18) alpha-glucose, (19) maltose, (20) ribose, (21) inosine, (22) homoserine, (23) aspartate, (24) glycerophosphocholine, (25) choline, (26) formate, (27) histidine, (28) leucine, (29) valine and (30) beta-glucose. The y-axis is indicative of peak intensity, which correlates to concentration.

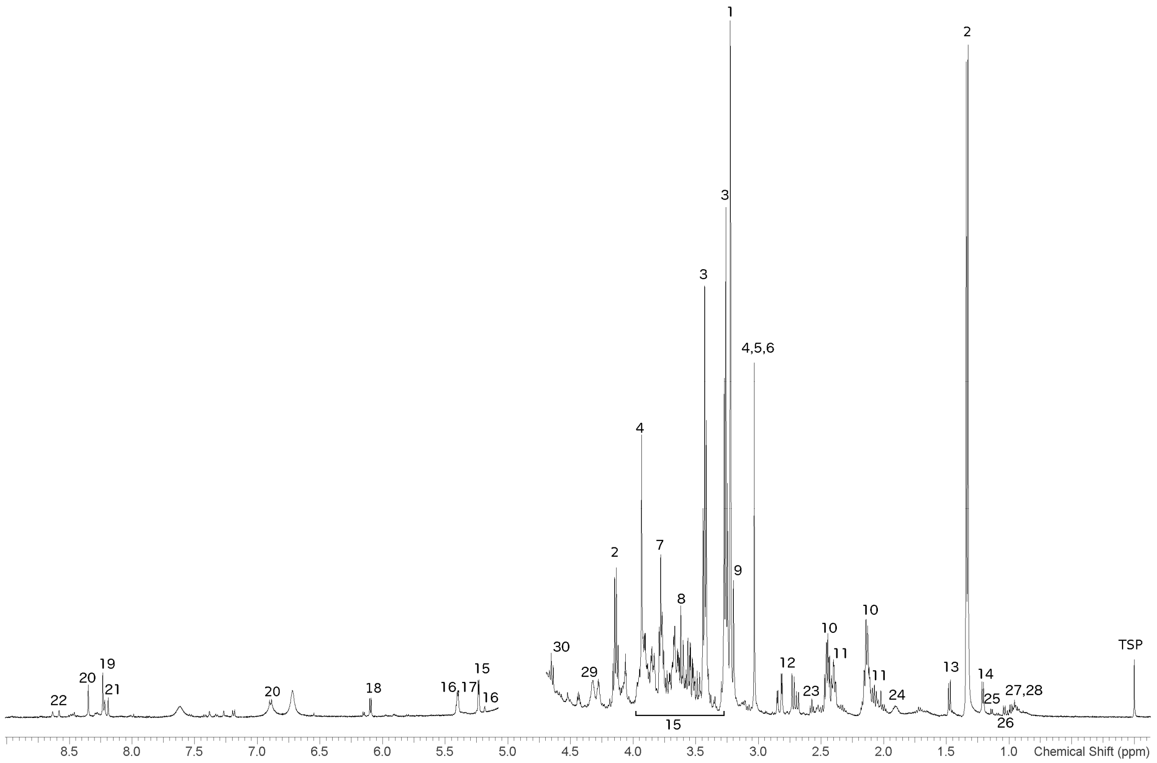

Figure S3. Representative ^{1}H-NMR spectrum of an aqueous extract of an individual hatchling loggerhead sea turtle (*Caretta caretta*) heart sample. The y-axis is indicative of peak intensity, which correlates to concentration. The water peak has been removed. Labeled peaks are as follows: (1) phosphocholine, (2) lactate, (3) taurine, (4) creatine (5) creatinine, (6) phosphocreatine, (7) glutathione, (8) glycine, (9) choline, (10) glutamine, (11) glutamate, (12) aspartate, (13) alanine, (14) 3-hydroxybutyrate, (15) alpha-glucose, (16) ribose, (17) glycogen, (18) adenosine triphosphate, (19) adenosine, (20) nicotinamide adenine dinucleotide (21) adenine, (22) adenosine diphosphate, (23) beta-alanine, (24) homoserine, (25) propylene glycol, (26) valine, (27) isoleucine, (28) leucine, (29) glycerophosphocholine and (30) beta-glucose.

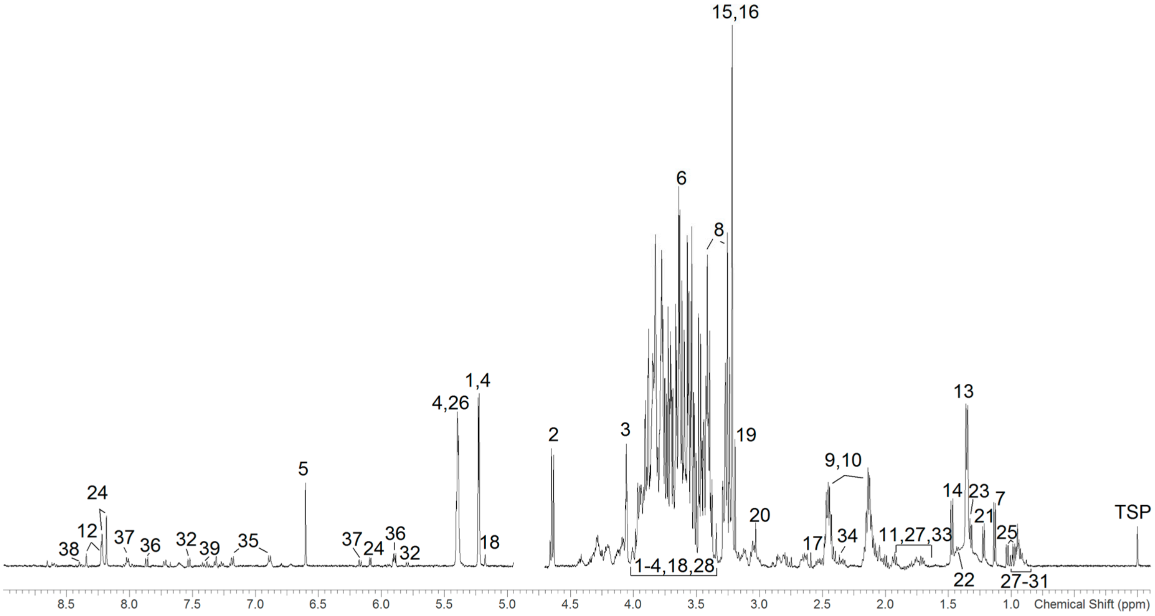

Figure S4. ^{1}H-NMR spectrum of the aqueous extract of combined control samples of hatchling loggerhead sea turtle (*Caretta caretta*) liver. The y-axis is indicative of peak intensity, which correlates to concentration. The water peak has been removed. Labeled peaks are as follows: (1) alpha-glucose, (2) beta-glucose, (3) myo-inositol, (4) maltose (5) histidine, (6) glycine, (7) unknown, (8) taurine, (9) glutamine, (10) glutamate, (11) glycylproline, (12) adenine, (13) lactate, (14) alanine, (15) glycerophosphocholine, (16) phosphocholine, (17) methylamine, (18) mannose, (19) choline, (20) creatine/creatine phosphate, (21) 3-hydroxybutyrate, (22) alanine dimer, (23) threonine, (24) inosine, (25) valine, (26) ribose, (27) leucine, (28) isoleucine, (29) alloisoleucine, (30) 2-hydroxybutyrate, (31) 2-hydroxyvalerate, (32) uracil, (33) arginine, (34) pyruvate, (35) tyrosine, (36) uridine, (37) cytidine, (38) adenosine triphosphate and (39) phenylalanine. The y-axis is indicative of peak intensity, which correlates to concentration. The water peak has been removed.

Table S1. List of metabolites identified in the aqueous extracts of skeletal muscle, heart and liver of hatchling loggerhead sea turtles (*Caretta caretta*) using 1-D and 2-D NMR experiments and their respective chemical shifts and multiplicity identified in our samples (d, doublet; dd, doublet of doublets; m, multiplet; s, singlet; t, triplet; tt, triplet of triplets)

**Metabolite**	**[1]H Chemical Shift (ppm) and Multiplicity from 1D and 2D spectra**	**[13]C Chemical Shift (ppm) from HSQC and/or HMBC spectra[a]**	**Tissue**
Organic acids/Osmolytes			
Acetate	1.92(s)	NA	S*
Carnosine	2.99 (dd), 3.11(dd), 3.24(dt), 4.53(m), 7.25(s), 8.57(s)	26.3, 53.9, 116.3, 133.7	S
Choline	3.20(s), 3.51(dd), 4.06(ddd)	56.5, 58.3, 70.1	S, H, L*
Creatinine	3.03(s), 4.07(s)	36.6, 56.6, 183.0	H*

Formate	8.44(s)	171.9	S
Glutathione	2.15(m), 2.54(m), 2.97(dd), 3.78(m), 4.2(q)	28.3, 29.0, 34.1, 46.1, 57.0, 58.4, 174.4, 176.7, 177.7, 178.9	H
Glycerophosphocholine	3.23(s), 3.56(dd), 3.66(m), 3.68(m), 3.91(m), 3.94(m), 4.33(bm)	56.9, 65.8, 65.8, 68.9, 73.7, 69.4	S, H, L*
Lactate	1.33(d), 4.15 (m)	18.7, 68.1,182.1	S, H, L*
Myo-inositol	3.27(t), 3.54(dd), 3.61(t), 4.07(t)	77.0	S, L*
Phosphocholine	3.22(s), 3.61 (t), 4.13(dddd)	56.5, 60.3, 68.2	S, H, L*
Energy compounds			
Creatine	3.04(s), 3.93(s)	39.8, 54.0	S, H, L*
Glucose (α & β)	3.22 β(dd), 3.53 α(dd), 3.76 β(m), 3.82 α(m), 4.65 β(d), 5.23 α(d)	91.5α, 95.3β	S, H, L*
Glycogen	3.83(m)	63.2, 72.1, 73.8, 74.3, 74.5, 75.5, 76.1, 79.4, 102.6	H*
Maltose	3.56(m), 3.88(dd), 3.93(d), 5.44(d)	99.0	S, L*
Mannose	3.98 (m), 5.16 (d)	93.5	L*
Ribose	4.21(m), 4.30(m), 4.40(q), 4.62(dd), 4.80(t), 6.13(d)	64.1, 65.2, 65.7, 65.9, 69.9, 71.7, 71.8, 72.6, 73.1, 73.4, 73.7, 78.0, 85.1, 85.9, 96.5, 99.0, 103.8	S, H, L*
Phosphocreatine	3.03(s), 3.89(s)	39.5, 66.4	S, H, L*
Amino acids			
Alanine	1.48(d), 3.84(m)	15.1, 19.7, 50.3, 174.2	S, H, L*
Alanine dimer	1.42(d)	19.7, 69.5, 174.4	L*
Alloisoleucine	0.95	20.2	L*
Aspartate	2.66(dd), 2.66(dd), 2.80(dd), 2.80(dd), 3.89(dd), 3.89(dd)	39.3, 55.1, 176.9, 180.2	S, H*
β -alanine	2.56(t), 3.18(t)	36.3, 39.5, 181.0	S, H*
Glutamate	2.45(m), 3.78(dd)	26.4, 53.0, 181	S, H, L*
Glutamine	2.11(m), 2.46(m), 3.75(t)	32, 180	S, H, L*
Glycine	3.57(s)	41.3	S, H, L*
Glycylproline	1.92 (m)	29.6	L*
Histidine	3.23(d), 3.25(d), 3.99(m), 6.64 (s), 7.08(s), 7.84(s)	134.6	S, L*
Homoserine	2.01(m), 2.16(m), 3.77(m), 3.85(dd)		S, H
Isoleucine	0.93(t), 1.02(d)	18.2, 14.4	H, L*
Leucine	0.84(d)	22.3	S, H, L*
Methylamine	2.62 (s)	30.8	L*
Phenylalanine	7.33 (m)	129.2	L*

Taurine	3.26(t), 3.43(t)	47.0, 35.1	S, H, L*
Threonine	1.31(d), 2.50(d), 4.21(m)	19.2	L*
Tyrosine	6.89(d), 7.19(d)	115.7, 131.6	L*
Valine	1.05(d), 2.12(m)	60, 29, 16	S, H, L*
Ketone bodies			
3-hydroxybutyrate	1.20(d), 2.31(dd), 2.40(dd), 4.15(m)	24.5	S, H, L*
2-hydroxybutyrate	0.94(t)	10.0	L*
2-hydroxyvalerate	0.97(t)	22.02	L
Pyruvate	2.37(s)	30.7	L*
Nucleosides, Nucleotides & Analogues			
Adenine	8.19(s), 8.25(s)	141.3, 159.2	H, L*
Adenosine	3.86(dd), 3.94(dd), 4.30(q), 4.44(dd), 4.81(s), 6.02(d), 8.12(s), 8.28(s)		H
Adenosine diphosphate	4.15(m), 4.16(m), 4.57(m), 5.94(m), 8.29(s), 8.54(s)		H
Adenosine triphosphate	4.21(m), 4.28(m), 4.40(m), 4.51(m), 4.62(t), 6.13(d), 8.24(s), 8.53(s)	144.8, 141	H, L*
Cytidine	6.09(d), 8.06(d)	87.06, 146.8	L*
Inosine	8.49(s), 8.50(s)	139.8, 134	S, L*
Inosine monophosphate	4.02(m), 4.35(m), 4.50(m), 6.13(d), 8.21(s), 8.55(s)		S
Nicotinamide adenine dinucleotide (NADH)	4.22(m), 4.35(m), 4.37(m), 4.34(dd), 4.50(m), 4.54(m), 6.03(d), 6.09(d), 6.12(d), 8.12(s), 8.03(m), 8.41(s), 8.84(d), 9.15(d), 9.33(s)		H
Uracil	5.80(d), 7.51(d)	148.2	L*
Uridine	5.88 (dd), 7.84(d)	100.4, 140.3	L*
Other			
Propylene glycol	1.14(d), 3.43 (dd), 3.53(dd), 3.87 (m)	18.8, 67.8, 68.3	S, H

[a] When metabolite concentration was low, ^{13}C cross peaks were not discernable.

[*] Confirmed using 2D +/- 1D ^{31}P NMR experiments, laboratory standards +/- predicted spectra (ACD SACD11/C+H Predictor and DB)(*).

Table S2. Chemical shift assignments and multiplicities of the ^{1}H-NMR and chemical shift assignments of the ^{13}C-NMR signals of the lipophilic extracts of hatchling loggerhead sea turtle (*Caretta caretta*) skeletal muscle, heart and liver. The signal number corresponds to the signal numbers in Figure 4. Key to multiplicity abbreviations: d=doublet, dd=doublet of doublets, dt=doublet of triplets, m=multiplet, s=singlet, t=triplet.

Signal	*^{1}H Chemical Shift (ppm) and multiplicity*[a]	*Type of Protons*	*^{13}C Chemical Shift (ppm) from HSQC and/or HMBC spectra*[a]	*Compound*
1	0.61 (s)	$-C\mathbf{H}_3$	11.4	Lathosterol
2	0.68 (s)	$-C\mathbf{H}_3$	12.2	Cholesterol
3	0.88 (t)	$-C\mathbf{H}_3$	14.4, 22.9	Fatty acids (except omega-3)
4	0.90 (t)	$-C\mathbf{H}_3$	13.9, 20.3	Cholesterol
5	0.98 (t)	$-C\mathbf{H}_3$	37.0	Fatty acids (omega-3)
6	1.00 (s)	$-C\mathbf{H}_3$	19.4	Cholesterol
7	1.02 (s)	$-C\mathbf{H}_3$	19.4	Esterified cholesterol
8	1.26, 1.28, 1.29 (m)	$-(C\mathbf{H}_2)_n-$	32.1, 29.7, 22.9	Fatty acids (except 20:5 omega-3 and 22:6 omega-3)
9	1.56, 1.60, 1.61 (m)	$-C\mathbf{H}_2-CH_2-COOH$	32.2, 28.0, 25.1	Fatty acids (except 20:5 omega-3 and 22:6 omega-3)
10	1.83 (m)	$-C\mathbf{H}_2-CH_2-COOH$	28.3, 37.3	Fatty acid (20:5 omega-3 and 22:6 omega-3)
11	2.01 (m)	$-C\mathbf{H}_2-CH=CH$	27.5, 40.0	Unsaturated fatty acids
12	2.28, 2.31 (dt)	$-C\mathbf{H}_2-COOH$	42.2, 38.7	Acyl group in triacylglycerides
13	2.32 (m,t)	$-C\mathbf{H}_2-COOH$	34.4	Acyl group in fatty acids (except 22:6 omega-3) and diacylglycerides
14	2.39 (t)	$-C\mathbf{H}_2-COOH$	23.0, 34.8	Fatty acid (22:6 omega-3)
15	2.81 (m)	$=CH-C\mathbf{H}_2-CH=$	26.2	Fatty acid (18:3 omega-3)
16	2.83 (m)	$=CH-C\mathbf{H}_2-CH=$	57.6, 25.9	Polyunsaturated fatty acids
17	3.31 (s)	$-N(C\mathbf{H}_3)_3$	54.9	Choline
18	4.15 (m)	$ROC\mathbf{H}_2-CH(OR')-C\mathbf{H}_2OR''$ $ROC\mathbf{H}_2-C\mathbf{H}OH-C\mathbf{H}_2OR'$	62.4	Glyceryl C1 protons of triacylglycerides and diacylglycerides
19	4.30 (dd,dd)	$ROC\mathbf{H}_2-CH(OR')-C\mathbf{H}_2OR''$ $ROC\mathbf{H}_2-CHOH-C\mathbf{H}_2OR'$	62.4	Glyceryl C1 protons of triacylglycerides and diacylglycerides
20	5.27 (m)	$ROCH_2-C\mathbf{H}(OR')-CH_2OR''$	69.3, 69.1	Glyceryl C2 protons in triacylglycerides
21	5.35 (m)	$-C\mathbf{H}=C\mathbf{H}-$	130.8, 121.7	Unsaturated fatty acids *cis*
22	5.37 (m)	$-C\mathbf{H}=C\mathbf{H}-$	130.8, 121.7	Unsaturated fatty acids *trans*
23	4.60 (m)	$-C\mathbf{H}-$	72.7	Cholesterol ester

[a] Data obtained from sample spectra (1D and 2D) and laboratory standards.

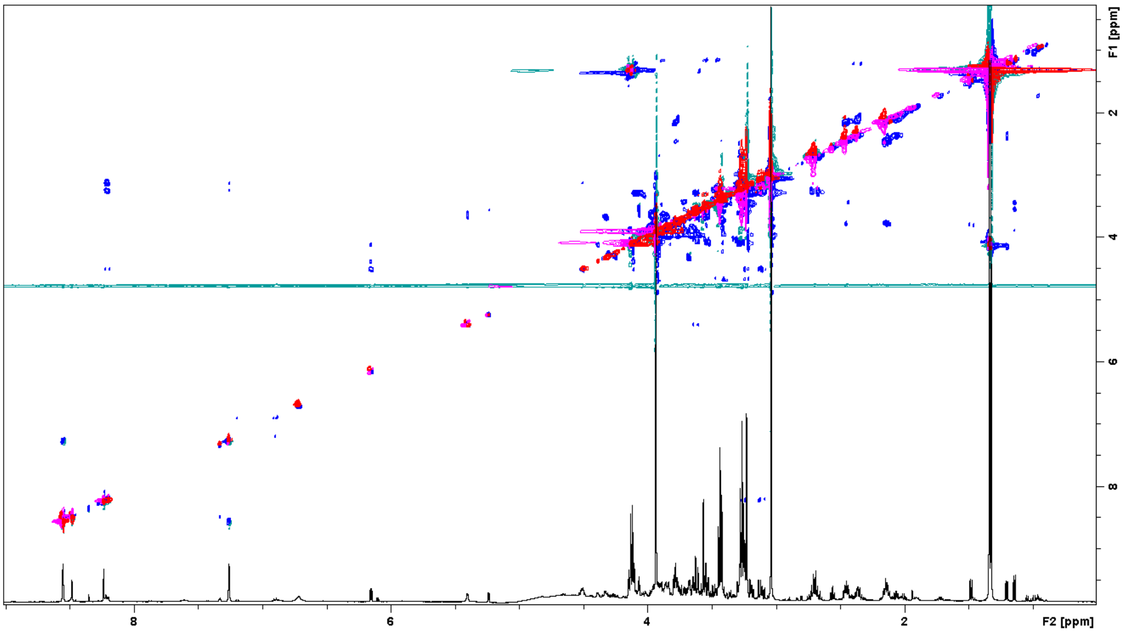

Figure S5. Combined (^{1}H-^{1}H)-NMR COSY (red) and (^{1}H-^{1}H)-NMR TOCSY (blue) spectra of the aqueous extract of combined control samples of hatchling loggerhead sea turtle (*Caretta caretta*) skeletal muscle. The 1-D ^{1}H-NMR spectrum (black) of this extract is on the x-axis.

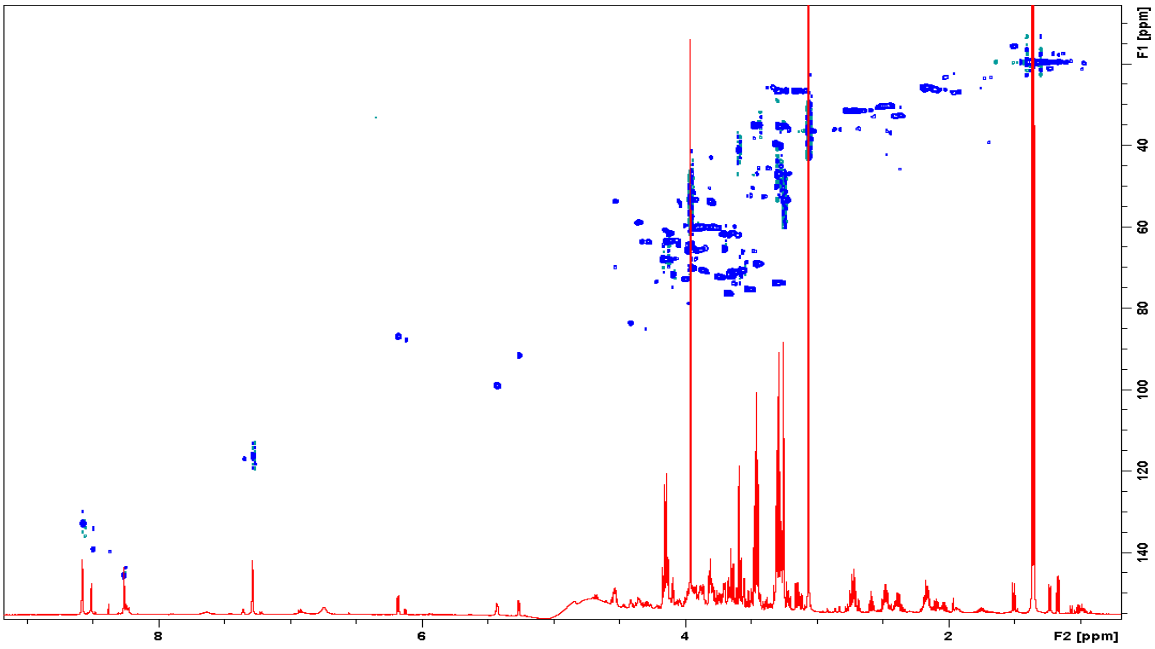

Figure S6. (^{1}H-^{13}C)-NMR HSQC spectrum (blue) of the aqueous extract of combined control samples of hatchling loggerhead sea turtle (*Caretta caretta*) skeletal muscle. The 1-D ^{1}H-NMR spectrum (red) of this extract is on the x-axis.

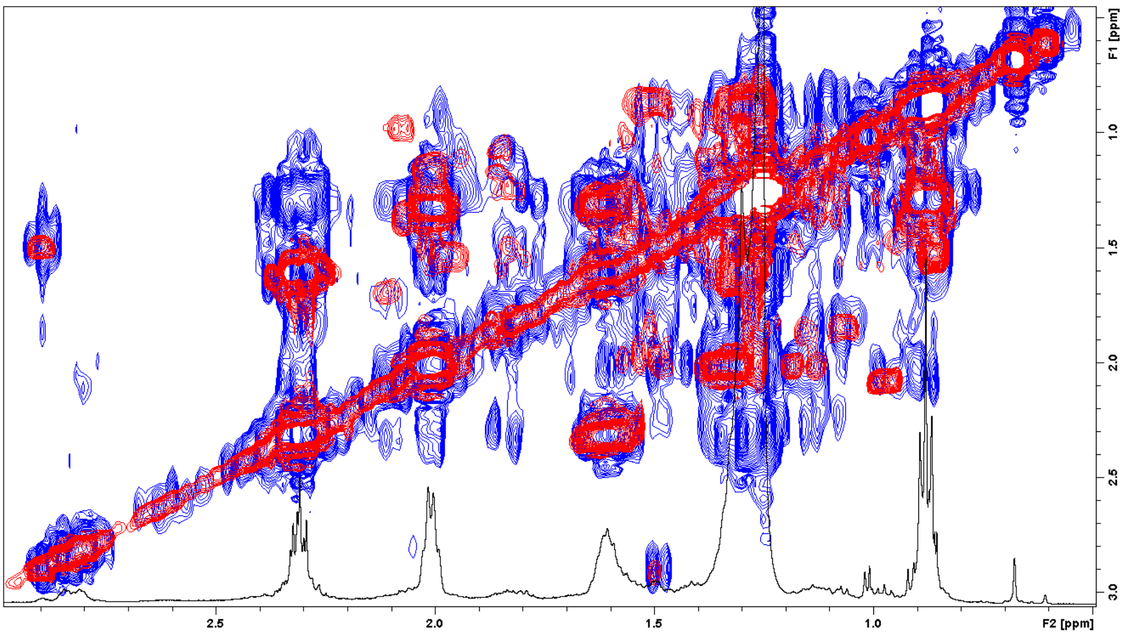

Figure S7. Combined (^{1}H-^{1}H)-NMR COSY (red) and (^{1}H-^{1}H)-NMR TOCSY (blue) of the lipophilic extract of combined control samples of hatchling loggerhead sea turtle (*Caretta caretta*) skeletal muscle**.** The 1-D ^{1}H-NMR spectrum (black) of this extract is on the x-axis.

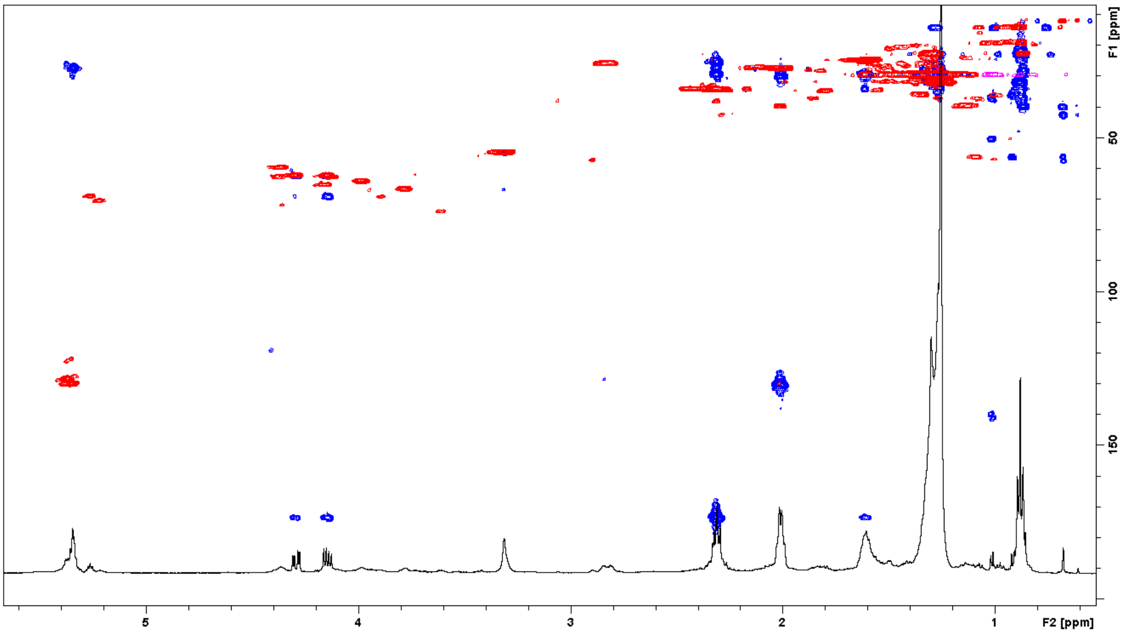

Figure S8. Combined **(**^{1}H-^{13}C)-NMR HSQC (red) and **(**^{1}H-^{13}C)-NMR HMBC (blue) of the lipophilic extract of combined control samples of hatchling loggerhead sea turtle (*Caretta caretta*) skeletal muscle. The 1-D ^{1}H-NMR spectrum (black) of this extract is on the x-axis.

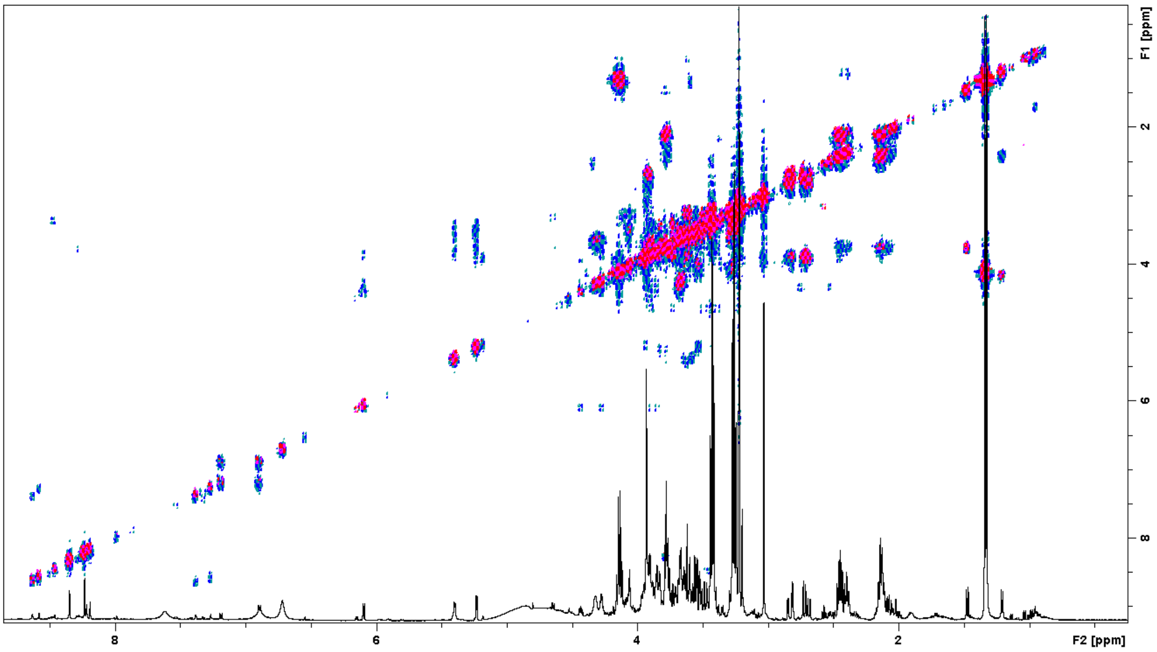

Figure S9. Combined (^{1}H-^{1}H)-NMR COSY (red) and (^{1}H-^{1}H)-NMR TOCSY (blue) spectra of an aqueous extract of a representative sample of hatchling loggerhead sea turtle (*Caretta caretta*) heart. The 1-D ^{1}H-NMR spectrum (black) of this extract is on the x-axis.

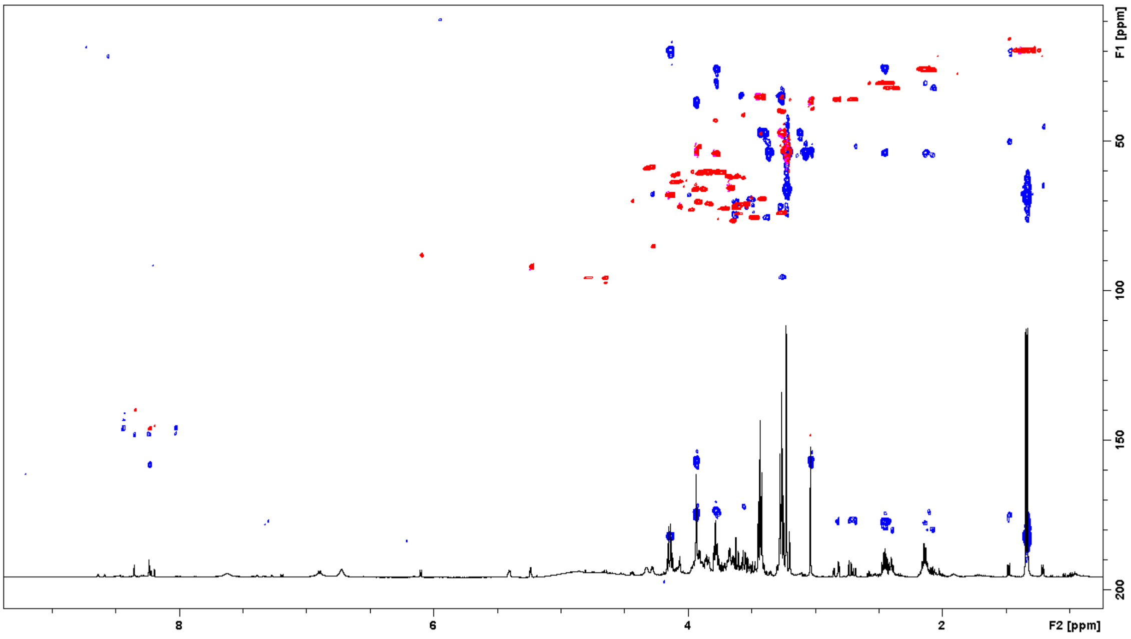

Figure S10. Combined (^{1}H-^{13}C)-NMR HSQC (red) and (^{1}H-^{13}C)-NMR HMBC (blue) spectra of an aqueous extract of a representative sample of hatchling loggerhead sea turtle (*Caretta caretta*) heart. The 1-D ^{1}H-NMR spectrum (black) of this extract is on the x-axis.

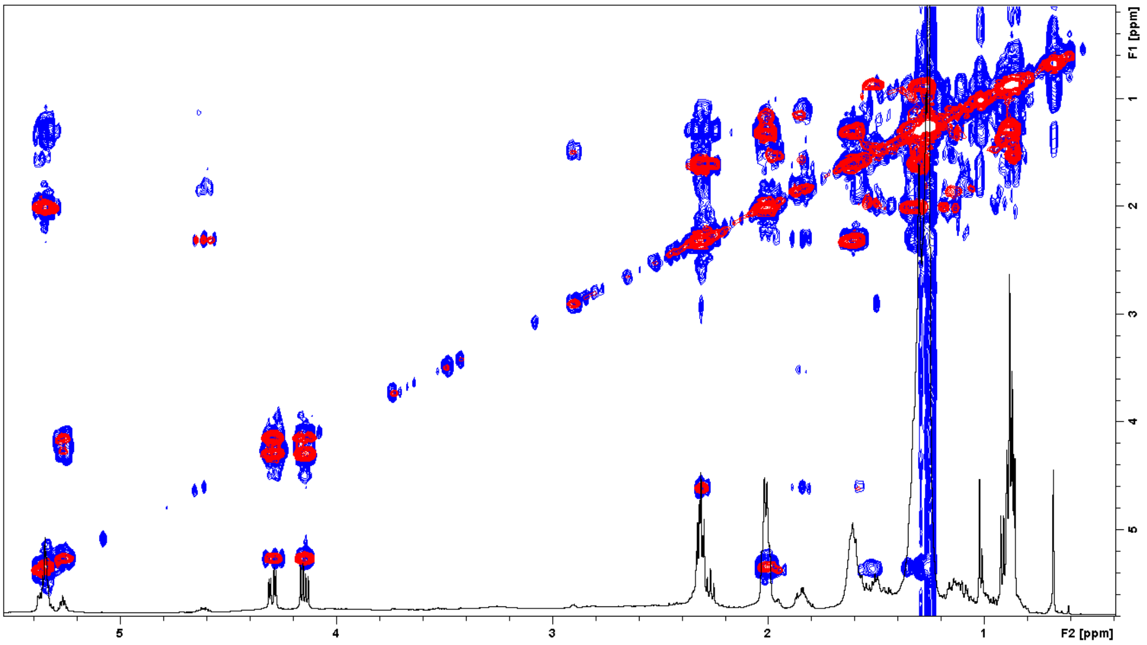

Figure S11. Combined (^{1}H-^{1}H)-NMR COSY (red) and (^{1}H-^{1}H)-NMR TOCSY (blue) spectra of a lipophilic extract of a representative sample (B45) of hatchling loggerhead sea turtle (*Caretta caretta*) heart. The 1-D ^{1}H-NMR spectrum (black) of this extract is on the x-axis.

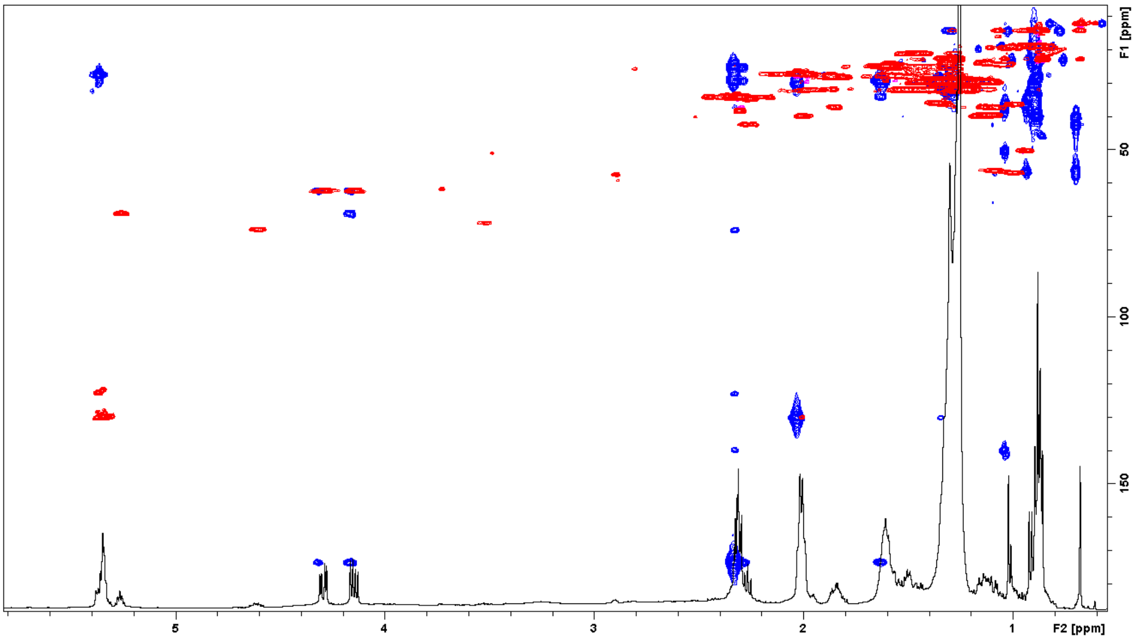

Figure S12. Combined (^{1}H-^{13}C)-NMR HSQC (red) and (^{1}H-^{13}C)-NMR HMBC (blue) spectra of a lipophilic extract of a representative sample (B45) of hatchling loggerhead sea turtle (*Caretta caretta*) heart. The 1-D ^{1}H-NMR spectrum (black) of this extract is on the x-axis.

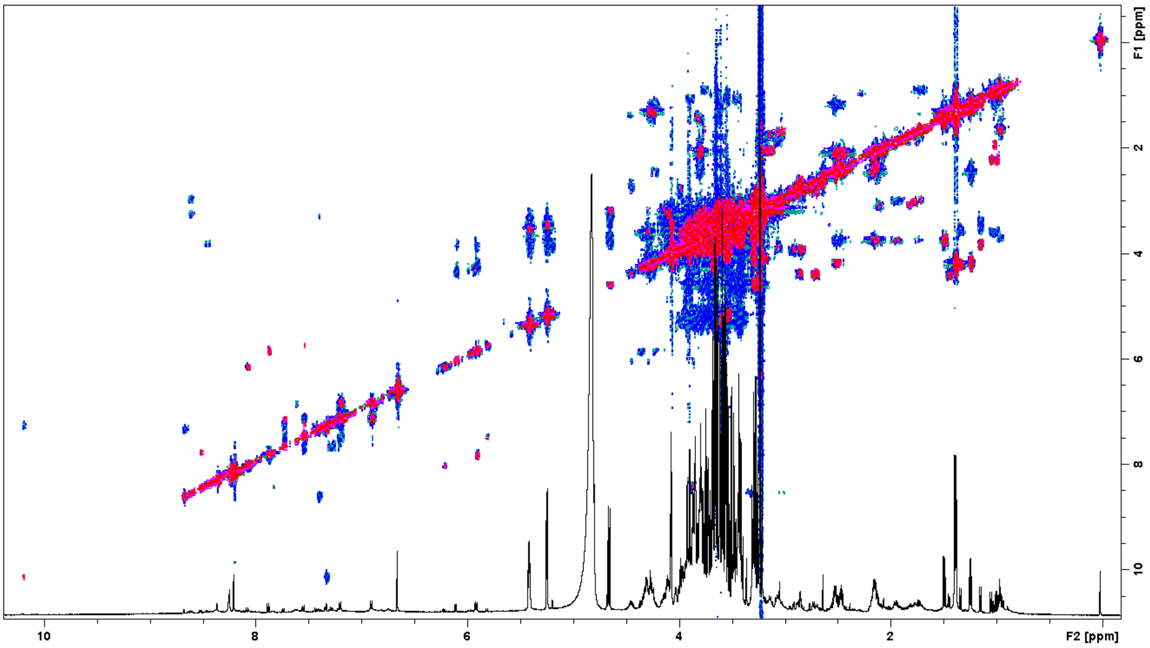

Figure S13. Combined (^{1}H-^{1}H)-NMR COSY (red) and (^{1}H-^{1}H)-NMR TOCSY (blue) spectra of an aqueous extract of a representative sample (B103) of hatchling loggerhead sea turtle (*Caretta caretta*) liver. The 1-D ^{1}H-NMR spectrum (black) of this extract is on the x-axis.

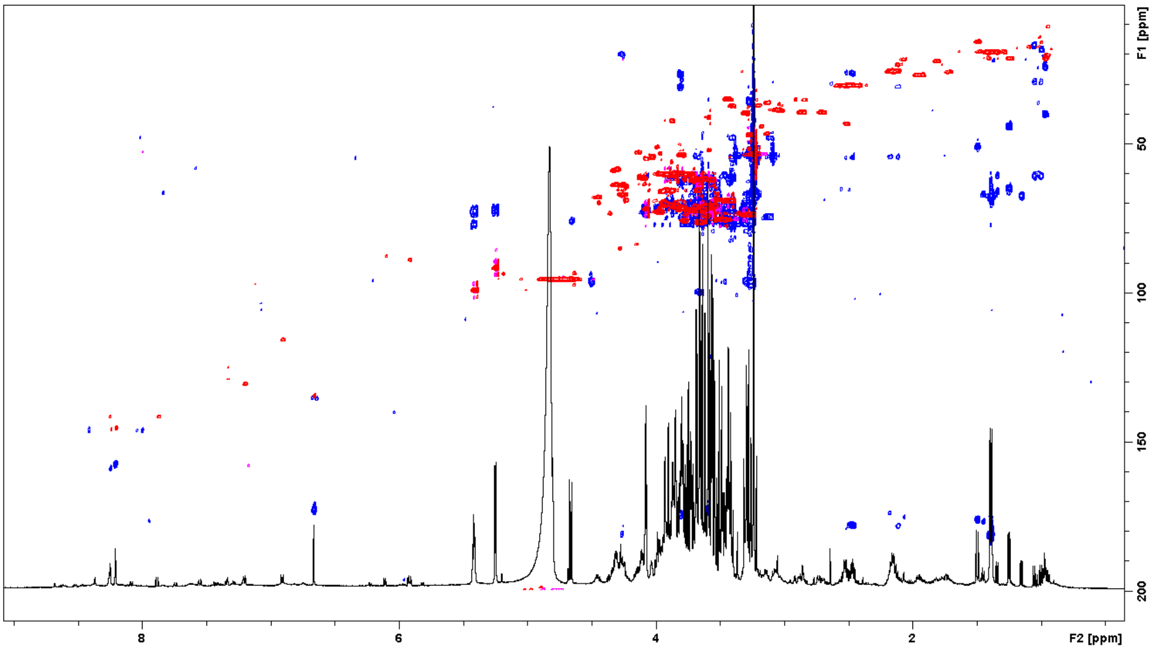

Figure S14. Combined (^{1}H-^{13}C)-NMR HSQC (red) and (^{1}H-^{13}C)-NMR HMBC (blue) spectra of an aqueous extract of a representative sample (B103) of hatchling loggerhead sea turtle (*Caretta caretta*) liver. The 1-D ^{1}H-NMR spectrum (black) of this extract is on the x-axis.

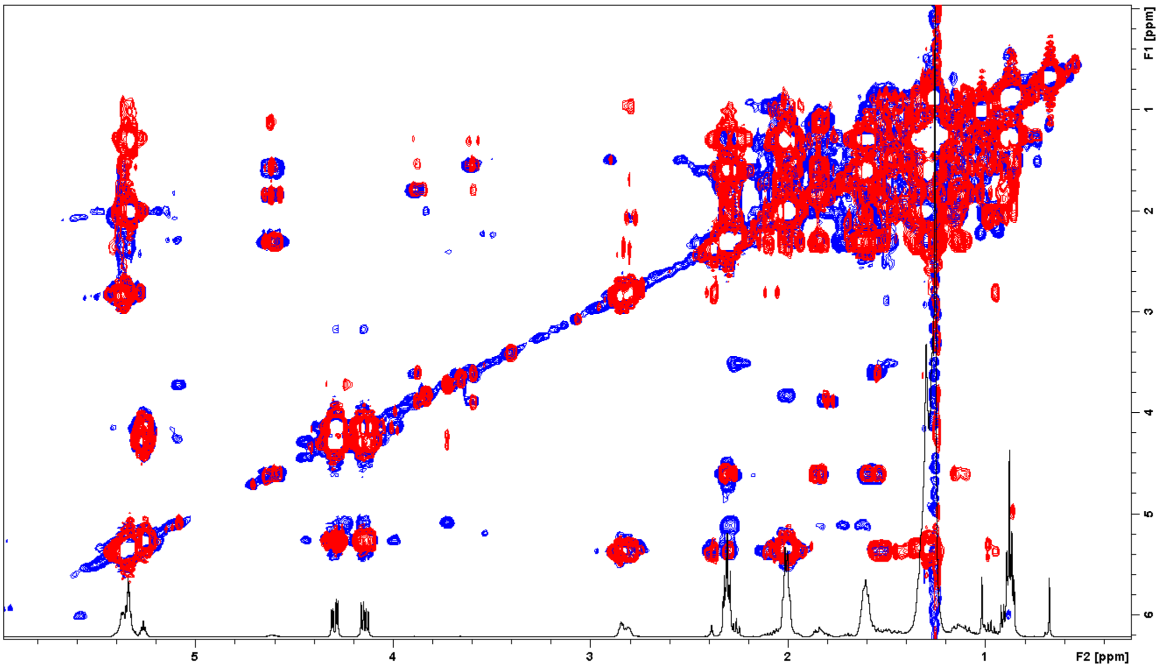

Figure S15. Combined (^{1}H-^{1}H)-NMR COSY (red) and (^{1}H-^{1}H)-NMR TOCSY (blue) spectra of a lipophilic extract of a representative sample (B103) of hatchling loggerhead sea turtle (*Caretta caretta*) liver. The 1-D ^{1}H-NMR spectrum (black) of this extract is on the x-axis.

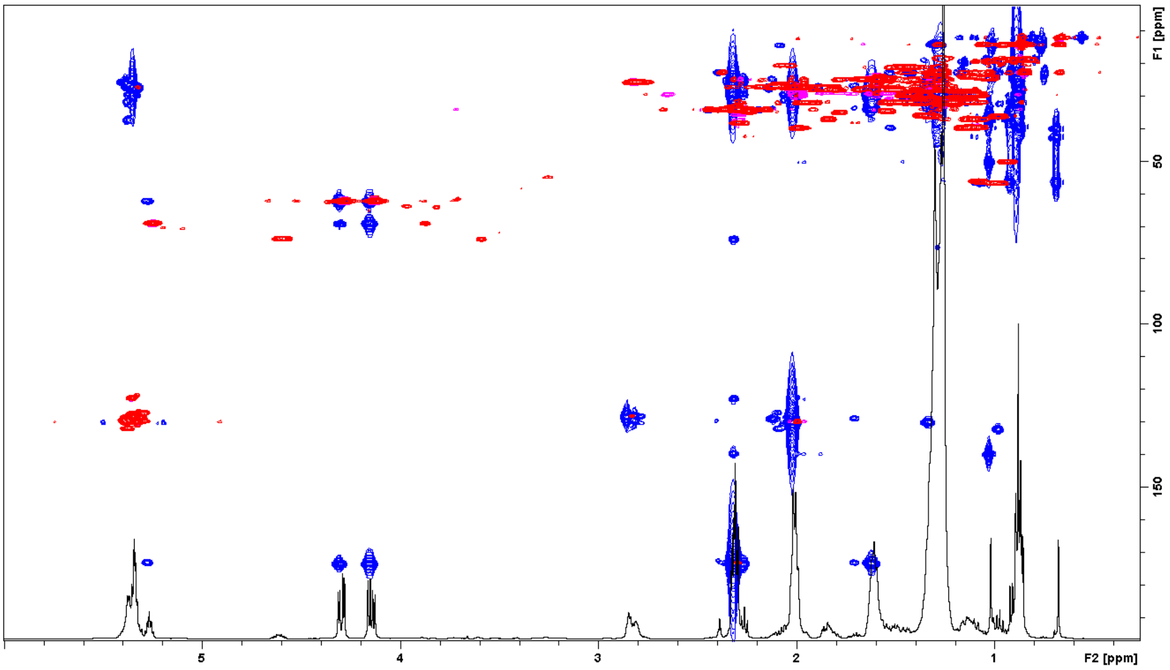

Figure S16. Combined **(**^{1}H-^{13}C)-NMR HSQC (red) and **(**^{1}H-^{13}C)-NMR HMBC (blue) spectra of a lipophilic extract of a representative sample (B103) of hatchling loggerhead sea turtle (*Caretta caretta*) liver. The 1-D ^{1}H-NMR spectrum (black) of this extract is on the x-axis.

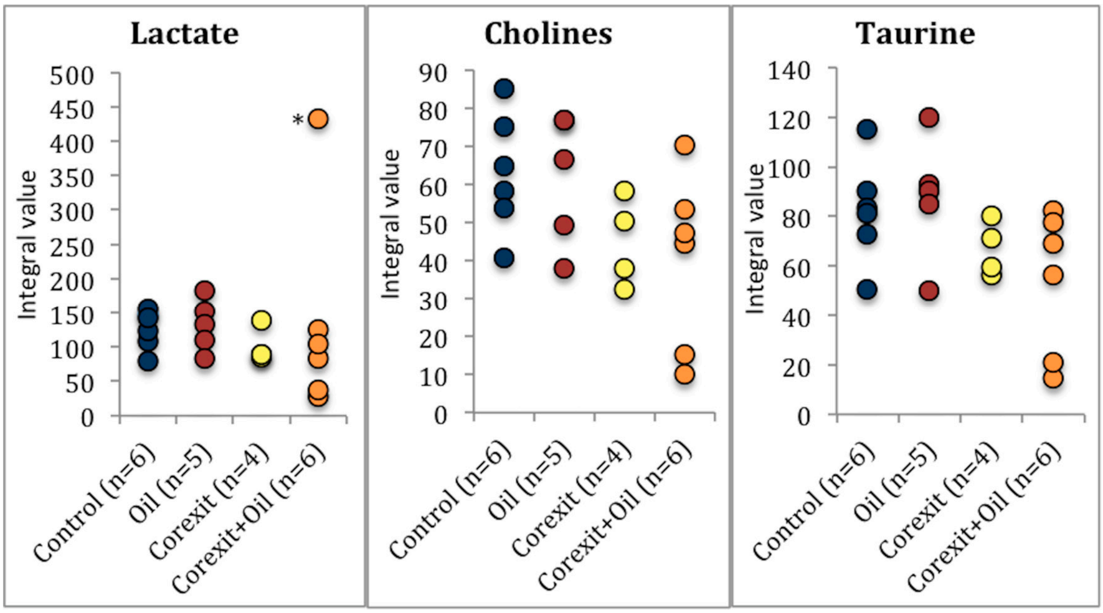

Figure S17. Individual value plots of the normalized and weighted integrals of lactate, cholines and taurine from the aqueous extracts of the loggerhead sea turtle (*Caretta caretta*) hatchling heart**.** Integral values are analogous to metabolite concentration. The treatment groups are labeled on the x-axis. For each metabolite, there were no significant differences among treatment groups (Kruskal Wallis tests: p = 0.080, p = 0.590, p = 0.166, respectively, α = 0.05). * Sample B45 from a hatchling exposed to crude oil and Corexit had a substantially higher lactate concentration than other samples. This hatchling died during the final processing.

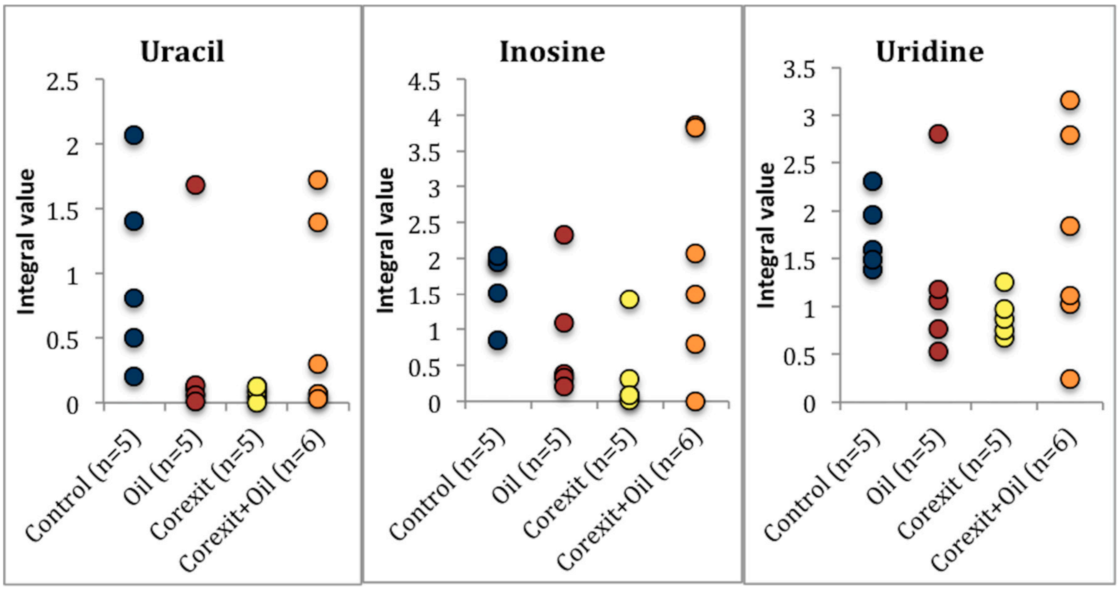

Figure S18. Individual value plots of the normalized and weighted metabolite integrals from the aqueous extracts of the loggerhead sea turtle (*Caretta caretta*) hatchling liver for which $p \leq 0.1$ in Kruskal Wallis tests, including uracil, inosine and uridine. Integral values are analogous to metabolite concentration. The treatment groups are labeled on the x-axis. For each metabolite, there were no statistically significant differences among treatment groups (Kruskal Wallis tests: p = 0.073, p = 0.080, p = 0.068, respectively; α = 0.05).

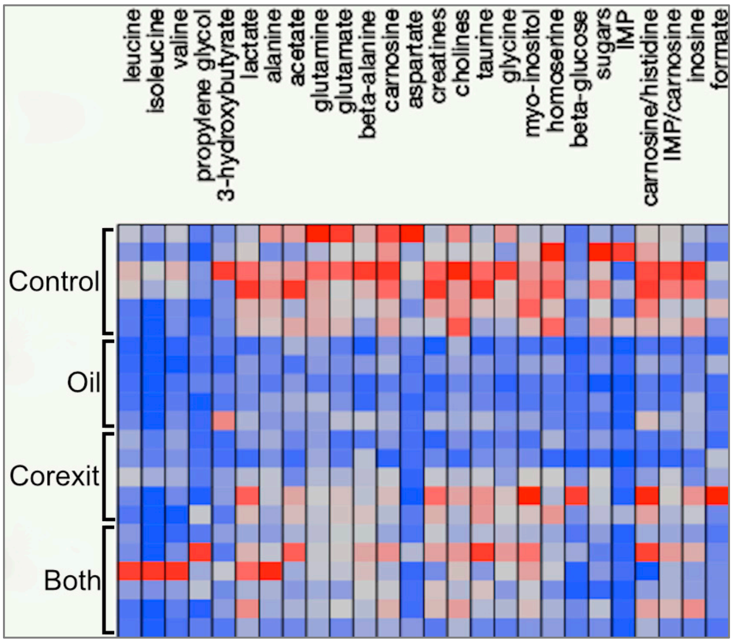

Figure S19. Heat map of normalized and weighted metabolite integrals identified in aqueous extracts of hatchling loggerhead sea turtle (*Caretta caretta*) skeletal muscle. The treatment groups are on the y-axis (Control, Crude Oil, Corexit, and Both Crude Oil and Corexit). There is a possible trend of skeletal muscle catabolism for most polar compounds detected in samples from hatchlings exposed only to crude oil.

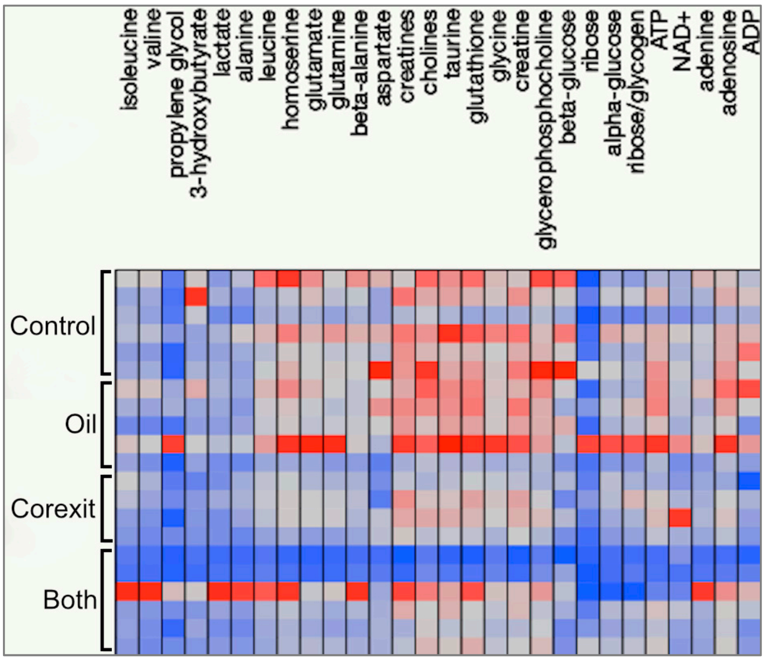

Figure S20. Heat map of normalized and weighted metabolite integrals identified in aqueous extracts of hatchling loggerhead sea turtle (*Caretta caretta*) heart. The treatment groups are on the y-axis (Control, Crude Oil, Corexit, and Both Crude Oil and Corexit).

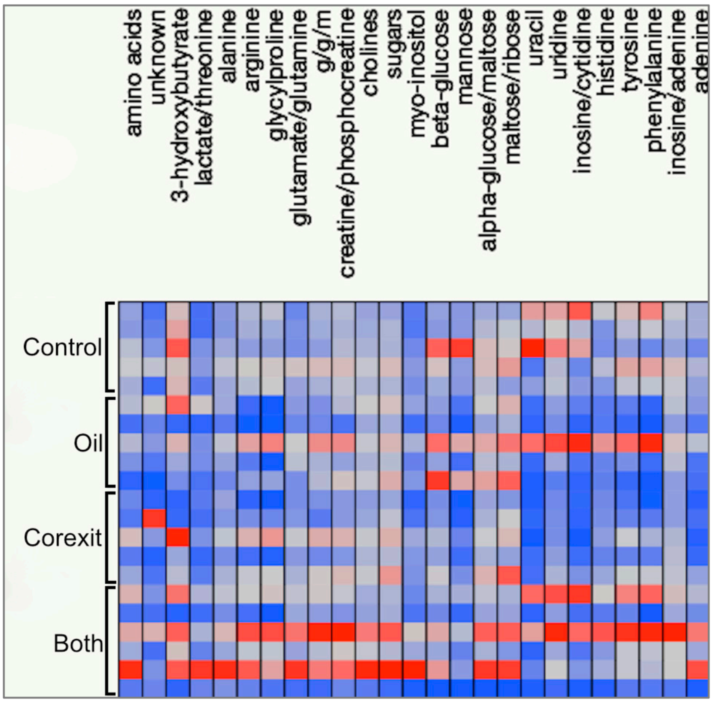

Figure S21. Heat map of normalized and weighted metabolite integrals identified in aqueous extracts of hatchling loggerhead sea turtle (*Caretta caretta*) liver. The treatment groups are on the y-axis (Control, Crude Oil, Corexit, and Both Crude Oil and Corexit). Key: (g/g/m) glutamate/glutamine/methylamine.

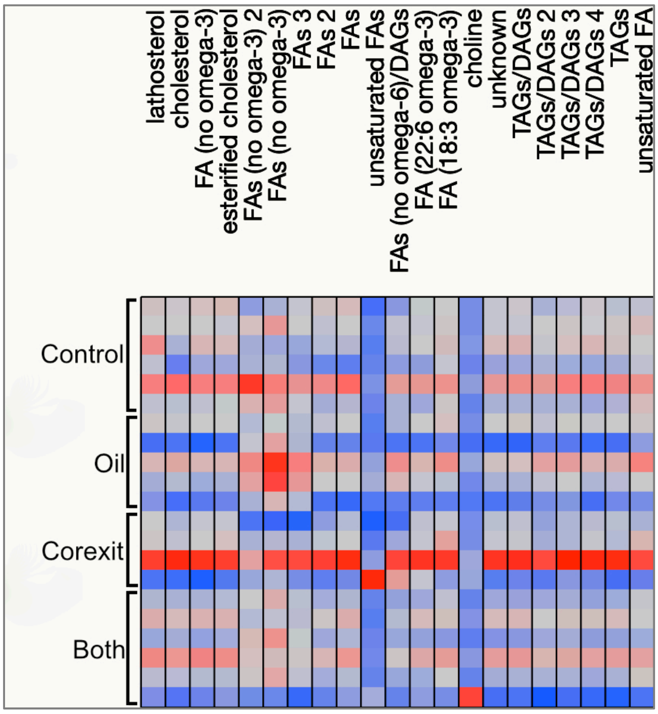

Figure S22. Heat map of functional group integrals from compounds identified in in skeletal muscle lipophilic extracts from hatchling loggerhead sea turtles (*Caretta caretta*). The treatment groups are on the y-axis (Control, Crude Oil, Corexit, and Both Crude Oil and Corexit). Key: (FA) fatty acid, (FAs) fatty acids, (DAGs) diacylglycerides, (TAGs) triacylglycerides.

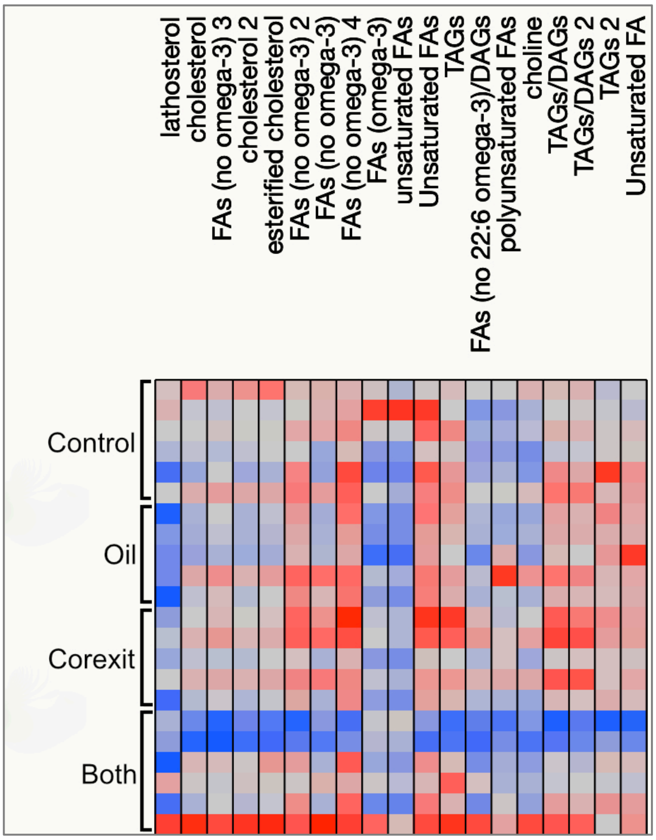

Figure S23. Heat map of functional group integrals from compounds identified in in heart lipophilic extracts from hatchling loggerhead sea turtles (*Caretta caretta*). The treatment groups are on the y-axis (Control, Crude Oil, Corexit, and Both Crude Oil and Corexit). Key: (FA) fatty acid, (FAs) fatty acids, (DAGs) diacylglycerides, (TAGs) triacylglycerides.

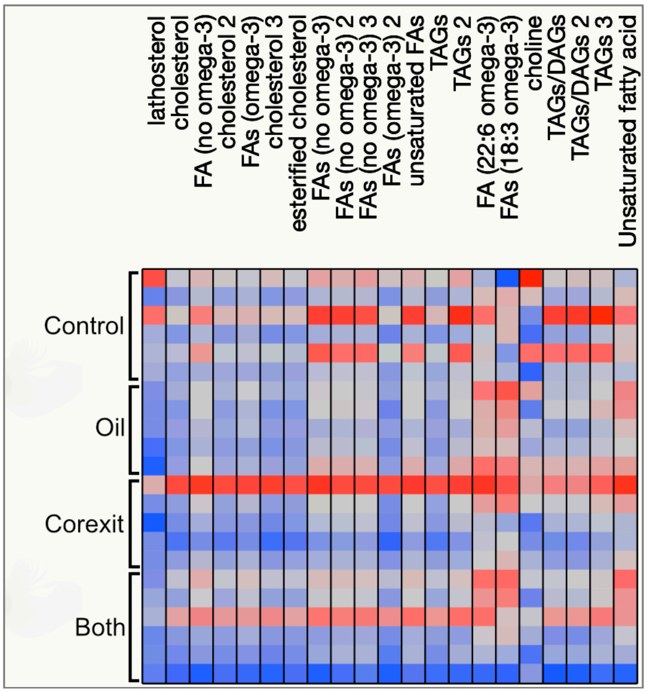

Figure S24. Heat map of functional group integrals from compounds identified in liver lipophilic extracts from hatchling loggerhead sea turtles (*Caretta caretta*). The treatment groups are on the y-axis (Control, Crude Oil, Corexit, and Both Crude Oil and Corexit). Key: (FA) fatty acid, (FAs) fatty acids, (DAGs) diacylglycerides, (TAGs) triacylglycerides.
